# Attitudes Toward Patient Safety in Operating Rooms: Cross-Cultural Adaptation and Validation of the French Version of the Operating Room Management Attitudes Questionnaire (ORMAQ)

**DOI:** 10.3390/healthcare14111465

**Published:** 2026-05-26

**Authors:** Mohamed Ayoub Tlili, Wiem Aouicha, Mouna Idoudi, Maali Haoues, Nikoloz Gambashidze, Hamdi Lamine, Maha Dardouri, Mohammad Alboliteeh, Sameer Alkubati, Bushra Alshammari, Oumaima Mohamed Ahmed Elalem, Nahed Moussa Saber, Matthias Weigl, Aziza Zakaria Ali

**Affiliations:** 1 Nursing Administration Department, College of Nursing, University of Hail, Hail 2440, Saudi Arabia; 2 Faculty of Medicine of Sousse, University of Sousse, Sousse 4002, Tunisia; 3 Higher School of Health Sciences and Techniques of Sousse, University of Sousse, Sousse 4054, Tunisia; 4 Institute for Patient Safety, University Hospital Bonn, Venusberg-Campus-1, 53127 Bonn, Germany; 5 Community Health Nursing Department, College of Nursing, University of Hail, Hail 2440, Saudi Arabia; 6 Medical Surgical Nursing Department, College of Nursing, University of Hail, Hail 2440, Saudi Arabia; 7 Maternity and Newborn Health Department, College of Nursing University of Hail, Hail 2440, Saudi Arabia; 8 Maternity and Newborn Health Department, Faculty of Nursing Beni-Suef, University of Beni-Suef, Beni-Suef 62511, Egypt; 9 Nursing Administration Department, Faculty of Nursing, Benha University, Benha 13511, Egypt

**Keywords:** patient safety, attitudes, operating rooms, cross-cultural validation

## Abstract

**Background**: The Operating Room Management Attitudes Questionnaire (ORMAQ) is widely used to assess operating room (OR) staff attitudes toward patient safety and teamwork across diverse contexts. However, no validated French version currently exists, limiting its use in francophone settings. This study aimed to translate and culturally adapt the ORMAQ into French and to evaluate its psychometric properties, while also reporting OR professionals’ attitudes explored during the validation process. **Methods**: A cross-sectional methodological study was conducted among OR professionals, including surgeons, anesthetists, anesthesia nurses, operating room nurses, and residents. The original ORMAQ was translated into French using a standardized forward–backward translation procedure and pretested with 20 OR professionals. Content and concurrent validity were examined. Reliability was assessed through internal consistency, test–retest reproducibility, and dimension-level consistency. Construct validity was examined using both exploratory and confirmatory factor analyses (CFA). **Results**: The overall response rate to the survey was 76.5% (n = 303). The French ORMAQ demonstrated good internal consistency, as evidenced by both Cronbach’s alpha (α = 0.842) and McDonald’s Omega (ω = 0.98). For the individual dimensions, reliability values ranged from 0.597 to 0.891 for alpha and from 0.75 to 0.89 for Omega. Test–retest analysis showed excellent reproducibility (ICC = 0.96; 95% CI: 0.92–0.98). Factor analyses supported the eight-factor structure, with the CFA confirming good model fit and meaningful item loadings across dimensions, with standardized loadings ranging from 0.40 to 0.83. **Conclusions**: The French version of the ORMAQ showed satisfactory psychometric properties. It represents a robust tool for assessing safety and teamwork attitudes among OR professionals in francophone countries.

## 1. Introduction

The Operating Room (OR) is recognized as a high-risk environment and one of the most complex workplaces in healthcare [1,2,3]. This complexity arises from demanding management protocols, sophisticated technologies, and the high level of coordination required to respond effectively to rapidly changing clinical conditions [3,4]. Surgical care is an essential component of healthcare, with over 200 million surgeries performed worldwide each year [4]. However, despite major advances in surgery and anesthesia, and the implementation of numerous national and international patient safety initiatives over the past decade, deficiencies in the quality and safety of surgical care remain a global concern [4].

Studies on the incidence of adverse events (AEs)—defined as unintended injuries or complications resulting from healthcare management rather than the patient’s underlying condition, potentially leading to prolonged hospitalization, disability, or death [5]—consistently show that most of these events are linked to surgical care and occur more frequently in ORs than in other hospital settings [2,3,4,6], with a reported median incidence of 40% of surgical procedures being associated with at least one adverse event (ranging from 27% to 75%) [7]. Importantly, over half of this harm is considered preventable [8].

The causes of AEs have been extensively examined in the literature, with human error recognized as a key contributing factor [9,10,11]. Beyond technical expertise, human factors play a pivotal role in ensuring patient safety, maintaining high-quality care, and reducing errors and AEs [9,10,11]. These factors encompass non-technical skills such as leadership, communication, teamwork, decision-making, and management of stress and workload [12]. Understanding underlying attitudes that shape these behaviors is therefore essential [13,14]. Assessing OR professionals’ safety attitudes helps to identify strengths and weaknesses within surgical teams, offering valuable insights into areas that require improvement [13,14]. Among the most effective approaches to describe such attitudes are questionnaire-based surveys of surgical staff [15].

In this context, Helmreich et al. adapted the Cockpit Management Attitudes Questionnaire (CMAQ) and the Flight Management Attitudes Questionnaire (FMAQ), originally developed for aviation safety [16,17], to introduce the Operating Room Management Attitudes Questionnaire (ORMAQ). This instrument includes 60 items designed to evaluate OR professionals’ attitudes toward teamwork and patient safety [18]. ORMAQ has been well-established since then and has been applied in surgical settings across various countries and specialties [15,19,20,21,22,23].

Actually, several instruments have been developed to assess patient safety culture and attitudes, such as the Safety Attitudes Questionnaire and the Hospital Survey on Patient Safety Culture [24]. While these tools are widely used and have demonstrated robust psychometric properties, they are primarily designed for general healthcare settings and may not fully capture the unique characteristics and specific dynamics of ORs, which are characterized by high complexity, time pressure, and tightly coordinated interprofessional teamwork.

In contrast, the ORMAQ is specifically grounded in human factors and crew resource management principles adapted from aviation, focusing on non-technical skills such as leadership, communication, decision-making, and error management in surgical teams. These dimensions remain central to contemporary patient safety frameworks and are consistently associated with improved team performance and patient outcomes [25,26,27,28].

Although the ORMAQ was originally developed several decades ago, its conceptual foundations remain highly relevant today, as modern patient safety approaches continue to emphasize the role of non-technical skills and human factors in preventing adverse events [29]. The growing global focus on surgical safety and teamwork, particularly in low- and middle-income countries, has further highlighted the need for context-specific and culturally adapted measurement tools [30,31,32].

Its diffusion across languages has not been uniform [33,34]. This may partly reflect the progressive integration of patient safety and human factors concepts into healthcare research and practice, particularly following landmark reports such as To Err Is Human [35]. In many healthcare systems, including francophone settings, earlier research efforts have predominantly relied on general safety culture instruments rather than context-specific tools focused on ORs [26]. This may have contributed to a delayed prioritization of instruments such as the ORMAQ in these contexts. The present study can therefore be seen as part of a more recent shift toward the targeted assessment of safety attitudes in high-risk clinical environments.

Despite these developments, validated instruments specifically tailored to OR environments remain limited in francophone settings. To date, no validated French version of the ORMAQ is available. In this context, the availability of a culturally adapted and psychometrically validated French version of the ORMAQ is both timely and necessary to support research, benchmarking, and quality improvement initiatives in francophone regions, including North Africa, where data on OR safety culture remain scarce.

Therefore, the present study aimed to translate and culturally adapt the ORMAQ into French, to validate its psychometric properties, and to report the attitudes of OR professionals collected during the validation process.

## 2. Methods

### 2.1. Study Design

A cross-sectional methodological study was conducted between February and April 2022, during which we established a consecutive translation and validation process using a stepwise procedure of Vallerand (1989) [36].

### 2.2. Study Population and Data Collection

The study was conducted in two public hospitals located in the region of Sousse, Tunisia, which together comprised 12 surgical departments and 36 ORs. The target population included all OR professionals (n = 396), namely surgeons, anesthetists, anesthesia nurses, OR nurses, and residents. Authorization to conduct the study was obtained from the chiefs of the participating departments before data collection.

The main sample consisted of OR professionals who voluntarily completed the French version of the ORMAQ for the assessment of construct validity and reliability (n = 303). To ensure methodological rigor, different subsamples were used for the various validation steps. A panel of five experts participated in the initial linguistic and content evaluation of the translated version. Twenty OR professionals pre-tested the translated version. Twelve experts participated in the content validity assessment.

The adequacy of the main sample size (n = 303) for factor analysis was assessed using established criteria. A minimum ratio of five participants per item was considered appropriate for the 60-item scale, corresponding to at least 300 participants [37,38]. In addition, sample sizes of 300 are generally regarded as adequate for factor-analytic studies [39]. The Kaiser–Meyer–Olkin (KMO) measure of sampling adequacy was calculated to further assess the suitability of the data for factor analysis.

For concurrent validity and test–retest reliability, smaller subsamples were used (n = 10 and n = 20, respectively), due to the limited availability of bilingual participants and the practical constraints of repeated measurements, while preserving a sufficiently large main sample for factor-analytic procedures.

### 2.3. The ORMAQ

The latest version of the ORMAQ consists of sixty attitude statements relating to eight themes: leadership–structure; confidence–assertion; information sharing; stress and fatigue; teamwork; work values; error; organizational climate [18,21]. Respondents indicated the extent to which they agree with each statement on a 5-point Likert scale consisting of disagree strongly (1), disagree slightly (2), neutral (3), agree slightly (4) and agree strongly (5).

### 2.4. Procedure

#### 2.4.1. Translation of the ORMAQ and Evaluation of the Preliminary Version

The questionnaire was translated from the original English-language version using the forward–backward translation method [36,40]. The original version of the instrument was translated into French by a first translator. Then, this French translation was given to a second translator to translate it back into English, without the latter being helped by the original English version and without being in contact with the first translator.

Then, five experts evaluated the translated version, in comparison with the original English version. According to Vallerand (1989) [36], this operation consists of two levels of analysis: the first level shows whether the items in French correspond linguistically to the English original ones, and the second checks the content of the statement. Careful comparison of the differences between the French version obtained and the difference between the original and the translated versions allowed identifying the problematic items. On this occasion, simple linguistic (semantic) corrections were made, keeping the desired original meaning of the original version.

#### 2.4.2. Pretest of the Translated Version

The questionnaire was submitted to 20 OR professionals of different grades. This step was not limited, as recommended by Vallerand (1989) [36], to completion of the questionnaire. Participants were also asked to evaluate the clarity of each item, and to encircle any ambiguous expression or item. At the end of this step of reformulation and clarification of the items, a final version of the ORMAQ in French language was obtained.

#### 2.4.3. Validity of the Questionnaire

*Content validity*

The assessment of the content validity of the translated ORMAQ was based on experts’ evaluation. The French version was provided to a panel of specialists in OR safety and quality of care, who were recruited based on their clinical and academic expertise (n = 12). Each expert rated item relevance on a four-point Likert scale ranging from ‘so irrelevant’ to ‘very relevant’. A score at each statement was made to determine the Content Validity Index (CVI) by dividing the number of statements scored 3 and 4 by the total number of statements (60 statements).

*Concurrent validity*

Concurrent validity, which refers to the degree to which the translated version produces results consistent with those of the original instrument when administered to the same participants [41], was examined by administering both the original English and the translated French versions to ten bilingual OR professionals. The degree of correlation between responses on the two versions was analyzed to determine the equivalence of the two forms.

*Construct validity (Exploratory and confirmatory factor analysis)*

Construct validity refers to the extent to which the instrument accurately reflects the underlying theoretical dimensions it is intended to measure [41]. To evaluate the construct validity of the French version of the ORMAQ, both exploratory and confirmatory factor analyses were conducted using the main survey results (n = 303). The exploratory factor analysis (EFA) was first used to explore the underlying factor structure of the questionnaire and to identify the dimensions that best represent the data. Based on the results of the EFA, a confirmatory factor analysis (CFA) was then performed to test the hypothesized eight-factor model and to verify the adequacy of the proposed structure. These analyses aimed to ensure that the translated instrument retained the conceptual framework and dimensional structure of the original version while demonstrating satisfactory psychometric properties.

#### 2.4.4. Reliability of the Questionnaire

Reliability was assessed by testing internal consistency of the questionnaire and its dimensions among the main study population (n = 303). To assess reproducibility, a test–retest was conducted. Twenty randomly selected OR professionals were asked to answer the questionnaire twice with a one-month interval between the test and the retest.

### 2.5. Statistical Analysis

For the concurrent validity, the Pearson correlation coefficient was used to assess the relationship between the English and French versions. Internal consistency of the questionnaire and its dimensions was assessed using both Cronbach’s alpha coefficient and McDonald’s Omega (ω), the latter being a robust indicator of internal consistency, with values ≥ 0.70 considered acceptable. Test–retest reliability of the 60 items was assessed by the one-way intra-class correlation coefficient (ICC type (2, 1)).

Prior to conducting the exploratory factor analysis, the adequacy of the correlation matrix was verified using the Kaiser–Meyer–Olkin (KMO) measure of sampling adequacy and Bartlett’s test of sphericity. A principal component analysis was conducted for the construct validity of the ORMAQ. For the basis of this analysis, the components were extracted with an eigenvalue of greater than 1. Varimax rotation with Kaiser normalization was selected, and the rotation converged in 9 iterations, with values below 0.30 being suppressed. All those analyses were conducted by using SPSS 20.0 (IBM-SPSS Statistics, Armonk, NY, USA).

The confirmatory factor analysis (CFA) was conducted using IBM SPSS Amos version 26.0 with maximum likelihood estimation on the full sample (n = 303). The hypothesized eight-factor oblique model reflected the theoretical structure of the ORMAQ as originally defined by Helmreich et al. [18], comprising the following latent constructs: Confidence–Assertion, Leadership Structure, Information Sharing, Errors/Procedural Compliance, Organizational Climate, Work Values, Stress and Fatigue, and Teamwork. All inter-factor correlations were freely estimated. Model fit was evaluated using multiple indices and conventional benchmarks as recommended by Hu and Bentler [42]: the Comparative Fit Index (CFI) and Tucker–Lewis Index (TLI) ≥ 0.90 (acceptable) and ≥0.95 (good fit), the Root Mean Square Error of Approximation (RMSEA) ≤ 0.08 (acceptable) and ≤0.06 (good fit), along with its 90% confidence interval, and the Standardized Root Mean Square Residual (SRMR) ≤ 0.08. Given the sensitivity of the chi-square statistic (χ^2^) to sample size and model complexity, the χ^2^/df ratio was also reported. Item-to-factor assignments followed the exploratory factor analysis (EFA) solution. Standardized factor loadings (λ) were examined for significance and magnitude, with values ≥ 0.40 considered acceptable. Modification indices were examined post hoc, and a limited number of residual covariances were allowed when both statistical relevance and theoretical justification (e.g., similarity in item wording within the same subscale) were met.

Descriptive analyses were first performed to summarize main survey participants’ sociodemographic and professional characteristics, as well as their responses to the ORMAQ items. Prior to descriptive analysis, the distribution of the data was assessed using skewness and kurtosis statistics to examine normality. Frequencies and percentages were calculated for categorical variables, while means, standard deviations (SD), minimum, and maximum were computed for continuous variables. For the presentation of attitudes toward teamwork and safety, response categories were regrouped into three categories to facilitate interpretation: “agree” (combining “strongly agree” and “agree”), “neutral,” and “disagree” (combining “disagree” and “strongly disagree”).

### 2.6. Ethical Considerations

Participation was voluntary, and participants were informed about the study objectives, procedures, and their rights prior to inclusion. Ethical approval was obtained from the Ethics Committee of the Faculty of Medicine of Sousse. Data collection was conducted anonymously, and strict confidentiality was maintained throughout all stages of the study, with no identifying information collected or stored. All data were handled and analyzed in accordance with established ethical standards for research involving human participants.

In addition, formal permission to translate, culturally adapt, and validate the questionnaire was obtained from the original developers of the ORMAQ prior to the initiation of the study.

## 3. Results

### 3.1. Study Population

A total of 396 OR professionals were invited to participate in the study, of whom 303 completed the questionnaire, resulting in a response rate of 76.5%. Most respondents belonged to nursing staff (63.0%, n = 191), and 96 (31.7%) had worked for 10 years or more in their specialty (Table 1). These participants formed the main sample used for the assessment of construct validity, reliability, and analysis of attitudes toward teamwork and safety.

### 3.2. Content and Concurrent Validity

For content validity evaluated by the panel of 12 experts, the CVI obtained by dividing the number of items that scored 3 and 4 (n = 49) by the total number of items (n = 60) was 0.82.

The concurrent validity, assessed by the subsample of ten bilingual OR professionals, revealed a strong concordance between the two versions of the questionnaires (r (ORMAQ English; ORMAQ French) = 0.971, *p* < 0.001). Additionally, an analysis of variance on the scores of different dimensions did not reveal significant differences between the two versions (Table 2).

### 3.3. Construct Validity

Construct validity was examined using the main survey sample (n = 303). Prior to exploratory EFA, the Kaiser–Meyer–Olkin (KMO) measure of sampling adequacy was 0.88, indicating the suitability of the correlation matrix. The EFA, using varimax rotation, converged in nine iterations and extracted eight factors, which collectively explained 58.66% of the variance. These factors grouped items into dimensions similar to those proposed in the original ORMAQ (Table 3).

**Figure 1:**
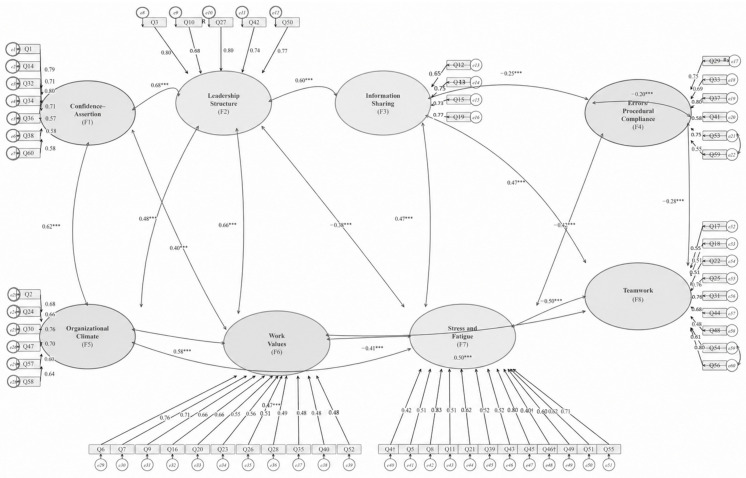
Final confirmatory factor analysis (CFA) model.

The CFA of the hypothesized eight-factor structure was conducted on the full sample (n = 303) using maximum likelihood estimation. The model demonstrated acceptable to good fit: χ^2^(1603) = 3426.18, *p* < 0.001; χ^2^/df = 2.14; CFI = 0.93; TLI = 0.91; RMSEA = 0.06 (90% CI: 0.05–.07); SRMR = 0.05. All standardized factor loadings were statistically significant (*p* < 0.001) (indicated by ***) and ranged from λ = 0.40 to λ = 0.83. Inter-factor correlations were statistically significant (*p* < 0.001) and ranged from low to moderate, consistent with an oblique factor structure. Two pairs of residuals were permitted to covary based on modification indices: between items Q53–Q59 (r = 0.28; Error/Procedural Compliance) and Q54–Q56 (r = 0.19; Teamwork). Composite reliability, assessed using McDonald’s Omega (ω), was excellent for the overall instrument (ω = 0.98) and ranged from 0.75 to 0.89 across subscales. The complete standardized CFA solution is presented in Figure 1. Items marked with † were retained despite loadings close to or slightly below the conventional threshold of 0.40, based on theoretical justification and consistency with the ORMAQ framework. In addition, items preceded by “R” were reverse scored before analysis; therefore, their loadings reflect the recoded metric.

### 3.4. Internal Consistency and Test–Retest Reliability

Cronbach’s alpha was 0.842 for the questionnaire and ranged from 0.597 to 0.891 for dimensions. McDonald’s Omega had a value of 0.98 for the whole questionnaire, and ranged between 0.75 and 0.89 for the different dimensions. As for the reproducibility of the questionnaire, assessed among twenty participants, the ICC for the ORMAQ had a value of 0.96 (0.92–0.98). The results of internal consistency and test–retest reliability are summarized in Table 4.

### 3.5. Attitudes Toward Teamwork and Safety Among ORs Professionals

Using the main survey sample (n = 303), descriptive analysis revealed that 66.7% of participants (n = 202) disagreed that doctors who encourage suggestions from operating theatre team members are weak leaders. Most professionals reported shared responsibility for prioritizing activities under high-workload conditions (87.5%, n = 265), and 80.2% (n = 243) indicated they would voice concerns if they perceived a problem. Effective information sharing requiring debriefing was endorsed by 89.8% of respondents (n = 272). Additionally, 54.8% (n = 166) disagreed that errors reflect incompetence, while 36.9% (n = 111) noted that procedures and protocols are not strictly followed in their ORs (Table 5).

## 4. Discussion

Ensuring patient safety in ORs is considered a priority worldwide, due to inherent complexities, the pace and nature of surgical work, and the rate of AEs occurring [2,3,4,6]. The description of the attitude of ORs professionals toward safety allows identifying strengths and weaknesses in safety practices, thus allowing a reliable assessment of the safety shortcomings that require special attention [13,14]. This study aimed to assess the reliability and validity of the French translation of the Operating Room Management Attitudes Questionnaire (ORMAQ). The validated French-ORMAQ will be useful for clinicians to obtain information about the safety and teamwork and for decision makers to improve healthcare services.

This study was conducted among 303 OR professionals meeting the inclusion criteria.

In general, sample sizes of 300 are regarded as adequate for factor-analytic studies [39]. The Kaiser–Meyer–Olkin (KMO) statistic of 0.88, obtained in our study, provides empirical confirmation of sampling adequacy for exploratory factor analysis. The smaller subsample sizes used for concurrent validity (n = 10) and test–retest reliability (n = 20) should be considered when interpreting these results. Although no universal consensus exists regarding minimum sample sizes for these specific validation steps, similar sample sizes have been reported in previous cross-cultural validation studies of OR safety instruments [43,44,45]. In the present study, these subsamples were partly constrained by the limited availability of bilingual professionals and the practical challenges associated with repeated data collection in clinical settings. Also, we aimed to avoid excessive participant burden and attrition, while preserving a sufficiently large main sample (n = 303) for the more statistically demanding analyses, particularly the exploratory and confirmatory factor analyses. While the obtained coefficients were strong (ICC = 0.96 for test–retest; r = 0.97 for concurrent validity), suggesting acceptable stability and equivalence, future studies with larger subsamples would help to further strengthen the robustness and generalizability of these findings.

To provide a more robust assessment of the factor structure, both exploratory and confirmatory factor analyses were conducted. While the use of independent samples is generally recommended to strengthen model validation [46], practical considerations related to sample size and model complexity must also be taken into account. Indeed, given the complexity of the present instrument (60 items across eight factors), methodological guidelines recommend relatively large sample sizes for stable CFA estimation, generally in the range of at least 200 participants [46,47]. In our case, splitting the total sample (n = 303) would have resulted in subsamples of approximately 150 participants, which may have been insufficient to ensure reliable and stable parameter estimation for a model of this complexity. Therefore, we opted to perform both EFA and CFA on the full sample, a practice that, while not ideal, has been adopted in similar validation studies when sample size constraints are present [44,48].

The whole translation/back-translation process led to the reformulation of certain sentences and the adoption of new terms such as “novice” instead of “junior”, in view to adapting the expressions to the French cultural context without changing the meaning. Similar changes were also reported in other studies [15,19,20,23].

The results of the concurrent validity analysis highlight congruence between the French and English versions of the ORMAQ. According to Vallerand [36], such high positive correlations support the adequacy of the translation process and the preservation of the intended meaning of the items. However, these findings do not necessarily imply full equivalence in dimensionality, which was further examined through exploratory and confirmatory factor analyses.

For the test–retest, the results showed good stability of the items’ responses as ICC obtained for the eight domains varied between 0.75 and 0.93. According to Vallerand, a correlation greater than 0.60 is usually desirable [36]. But according to Nunnally, as well as Streiner and Norman, the acceptable level of correlation must be situated above 0.70 [37,49]. Actually, a problem with test–retest reliability of instruments used in health care may be related to the fact that the perceptions of healthcare workers change over time, and sometimes even over a short period. The attitude, knowledge and skills of health practitioners may be affected by experiences between the test and the retest, which would make a measurement less reliable than it actually is [50].

For the internal consistency, although the overall Cronbach’s alpha of the instrument was satisfactory, two subscales (“error/procedural compliance” and “organizational climate”) showed values below the commonly accepted threshold of 0.70, suggesting relatively weaker internal consistency for these dimensions when assessed under the assumptions of this coefficient. This finding may be partly related to the limited number of items within these subscales and the potential multidimensionality of the underlying constructs. It is also important to note that Cronbach’s alpha assumes tau-equivalence, an assumption that may not always be met in multidimensional scales, potentially leading to underestimation of reliability. In this regard, McDonald’s Omega coefficients for these subscales reached acceptable levels (ω = 0.75), providing a more robust estimate of internal consistency [51,52]. Taken together, these results suggest that while these subscales remain usable, their interpretation should be made with caution. Both indices should be considered jointly when evaluating reliability, and further refinement and validation in larger samples may help improve their psychometric properties.

Based on the factor analysis of the French-ORMAQ, 60 items in the scale were divided into eight factors. Thus, there were no differences with the latest version of ORMAQ regarding item classification in domains [21].

To provide a more rigorous test of the theoretical model, a Confirmatory Factor Analysis (CFA) was conducted on the French ORMAQ. The model was evaluated with multiple indices to ensure a robust assessment. The results indicated an acceptable to good fit with the sample data. Furthermore, the Standardized Root Mean Square Residual (SRMR) was 0.05, also indicating a good fit. The CFA offered strong support for the hypothesized eight-factor structure, confirming that the items loaded significantly onto their respective latent constructs. The model fit indices indicated an acceptable to good fit with the sample data, with a Comparative Fit Index (CFI) of 0.93 and a Root Mean Square Error of Approximation (RMSEA) of 0.06, which falls below the conventional threshold of 0.08 for a good fit [42,47]. All standardized factor loadings were statistically significant (*p* < 0.001) and ranged from 0.40 to 0.83, demonstrating that each item was a meaningful indicator of its intended dimension. While some residual correlations were observed, the overall model structure was upheld, indicating that the French version successfully replicates the complex, multi-dimensional framework of the original ORMAQ [18]. This finding is crucial as it moves beyond merely discovering a structure (as EFA does) to actively testing and confirming the pre-defined theory in a new cultural context [46].

The confirmatory factor analysis revealed the inclusion of a limited number of correlated error terms, introduced based on modification indices to improve model fit. These correlated residuals likely reflect shared variance not fully captured by the latent constructs, potentially due to similarities in item wording or closely related content, a pattern commonly reported in large psychometric instruments [46].

While the inclusion of these correlated errors improved model fit indices, they should be interpreted with caution, as they may indicate partial redundancy between certain items. Nevertheless, the overall factor structure remained stable and consistent with the theoretical model of the ORMAQ, supporting its construct validity in the French context. Future studies should examine whether this structure can be replicated without the need for such modifications, ideally using independent samples. Inter-factor correlations further highlighted the relationships between the underlying dimensions. All correlations were positive and statistically significant, with most falling within the low to moderate range, supporting the interpretation of the subscales as distinct yet related constructs. However, higher correlations (r ≥ 0.60), particularly between Confidence–Assertion and Leadership Structure, Work Values and Leadership Structure, and Teamwork and Confidence–Assertion, suggest a degree of conceptual proximity between these domains. This overlap is theoretically plausible, as leadership, assertiveness, and teamwork behaviors are closely intertwined in high-risk clinical environments. These findings point toward the potential relevance of higher-order factor structures, which could be explored in future research to better capture the hierarchical organization of these interrelated competencies.

The satisfactory model fit affirms that the concepts of teamwork and safety attitudes, as conceptualized by Helmreich et al., are structurally valid and are measured equivalently by the French adaptation, thereby solidifying its construct validity for use in francophone ORs [18,46].

For the descriptive results related to items’ dimensions, we found that attitudes towards leadership, teamwork and information sharing were positive. However, the results reflected a negative attitude towards the dimensions stress and fatigue, procedural errors/conformities and organizational climate. The fear of speaking out when something does not seem to be right and the existence of a culture of blame and punishment for error seem to be recurring problems in ORs. Indeed, more than one-third of participants indicated that procedures and protocols are not strictly followed in their ORs. This finding is particularly important, as adherence to standardized protocols is a cornerstone of patient safety in surgical settings [53,54]. Similar observations have been reported in previous studies, where deviations from established procedures were associated with increased risk of adverse events and reflect underlying challenges related to safety culture, workload, and team communication [55,56,57]. In this context, the present results highlight the persistence of gaps between recommended practices and actual clinical behavior, emphasizing the need for targeted interventions to improve compliance, reinforce team accountability, and promote a culture of safety within OR teams.

Although the present study did not directly examine the relationship between safety attitudes and patient outcomes, this link is well established in the literature [24,58,59]. Previous studies have shown that positive safety attitudes, effective teamwork, and strong non-technical skills are associated with improved clinical outcomes and reduced adverse events [24,58,59]. In this perspective, the validated French ORMAQ provides a valuable foundation for future research exploring these associations in francophone surgical settings. The descriptive findings can also be interpreted in light of broader theoretical frameworks on safety culture and non-technical skills in surgical settings. The less favorable attitudes observed in domains such as stress, fatigue, and organizational climate are consistent with previous research highlighting the influence of workload, hierarchical dynamics, and communication barriers on team functioning and safety performance. Similarly, concerns related to error reporting and procedural compliance reflect well-documented challenges associated with blame culture and reluctance to speak up in ORs. These results suggest that the ORMAQ not only measures attitudes but also captures underlying cultural and organizational dynamics that may directly influence safe clinical practice.

Several studies found significant differences between nurses and surgeons [15,58,59,60]. For example, in a sample of professionals in Cyprus, nurses showed a higher positive perception than doctors regarding job satisfaction, stress recognition, and perceptions of management domains [58]. Also, another study revealed that nurses working in surgical units had a positive attitude toward patient safety, and previous training on patient safety significantly improved their attitude scores [59]. Kwon et al. showed that differences exist between doctors and nurses regarding teamwork climate, working conditions, perception of management and the recognition of stress [60]. In addition, on the performance of surgical time-out, nurses showed higher scores on way of counting, while doctors showed higher scores on the time-out procedure itself [60]. Interestingly, doctors believed they actively cooperated with the nurses, while nurses believed they did not receive cooperation [60].

### 4.1. Limitations

This study has several limitations that should be acknowledged. First, although the sample included professionals working across 36 ORs in 12 surgical units, all were located within a single Tunisian region (Sousse). Consequently, while the sample was heterogeneous in terms of professional role, age, gender, and years of experience, it may not fully represent the diversity of OR environments and safety cultures across Tunisia or other francophone contexts. Expanding future research to include multiple institutions and regions, particularly from different countries, would strengthen the generalizability and cultural robustness of the French version of the ORMAQ. Also, the study design was cross-sectional. In addition, the subsample sizes used for concurrent validity and test–retest reliability were relatively small, which may limit the precision and generalizability of these estimates. Although this is common in cross-cultural validation studies, particularly when bilingual participants are required, future studies should aim to include larger subsamples to strengthen the robustness of these validation steps.

Therefore, while the psychometric analyses support the reliability and validity of the questionnaire, and although test–retest analysis confirmed the temporal stability of the French ORMAQ, the study did not assess its responsiveness to change. Future longitudinal or interventional research is needed to determine whether the instrument can detect changes in safety attitudes following specific training or organizational interventions.

Further methodological consideration should be noted. The exploratory and confirmatory factor analyses were conducted on the same sample, which may increase the risk of overfitting and limit the generalizability of the findings. Future studies should validate the factor structure using independent samples. A limited number of correlated error terms were included in the CFA model to improve fit, which may indicate potential item redundancy and should be further examined in future validation studies. In addition, two subscales showed lower Cronbach’s alpha values, although Omega coefficients suggested acceptable reliability; these dimensions should therefore be interpreted with caution.

### 4.2. Study Implications

The validation of the French version of the ORMAQ provides a psychometrically sound tool for evaluating safety and teamwork attitudes among francophone OR professionals. Beyond its methodological contribution, this study provides meaningful added value by extending the applicability of the ORMAQ to a new linguistic and cultural context. The validation of the French version not only supports the robustness of the original theoretical framework but also offers empirical insight into safety attitudes among OR professionals in a North African setting, a region that remains underrepresented in patient safety research. By enabling context-specific assessment of non-technical skills in surgical teams, this work contributes to ongoing efforts to strengthen safety culture and improve patient outcomes in diverse healthcare environments.

Beyond its role as a measurement tool, the French ORMAQ can support targeted improvement strategies within OR teams. By identifying specific areas of weakness, such as communication patterns, perceptions of error, or organizational climate, it can guide tailored interventions and inform training initiatives focused on non-technical skills. When used longitudinally, it may also contribute to monitoring changes in safety attitudes following organizational or educational interventions.

At the educational level, the instrument can be integrated into professional training and continuing education programs to strengthen awareness of non-technical skills and their impact on patient outcomes. By revealing attitude differences across professional groups, it may guide the design of tailored educational strategies that promote interprofessional collaboration and a shared culture of safety.

From a research perspective, the validated French ORMAQ facilitates future comparative and cross-cultural studies, enhancing understanding of how contextual and cultural factors influence safety attitudes in surgical settings.

Finally, the results highlight persistent challenges related to stress, fatigue, and procedural adherence. Incorporating the ORMAQ into institutional assessment frameworks can help hospitals design evidence-based interventions that foster open communication, reduce workload-related risks, and promote a non-punitive approach to error reporting, ultimately supporting sustainable improvements in patient safety culture.

## 5. Conclusions

The French version of the Operating Room Management Attitudes Questionnaire (ORMAQ) demonstrated solid psychometric properties, confirming its validity and reliability for assessing safety attitudes among OR professionals. Through rigorous translation and cross-cultural adaptation, the instrument maintained the conceptual and dimensional integrity of the original version while achieving satisfactory construct validity, internal consistency, and test–retest reliability. This validated tool provides a reliable means of evaluating key components of safety culture, such as teamwork, communication, leadership, stress management, and organizational climate, within Francophone surgical settings. Its use may help identify areas for improvement and guide interventions aimed at strengthening team dynamics and patient safety.

Overall, this study offers a valuable contribution to the assessment of safety culture in Francophone healthcare contexts and supports the broader goal of enhancing safety performance and resilience in OR environments. Future research should aim to replicate these findings in larger, multicenter samples and explore the instrument’s sensitivity to change following targeted safety interventions.

## Figures and Tables

**Table 1:** Characteristics of participants (main sample).

Characteristics	Frequency (n)	Percentage (%)
***Total***	*303*	*100*
**Gender**		
Male	110	36.3
Female	193	63.7
**Age**		
20–30	65	21.4
31–40	169	55.8
41–50	43	14.2
>50 years	26	8.6
**Professional grade**		
Medical staff	112	37.0
Nursing staff	191	63.0
**Work experience in the actual OR**		
<6 years	165	54.5
≥6 years	138	45.5
**Work experience**		
<5 years	122	40.2
5–10 years	85	28.1
≥10 years	96	31.7
**Participation in risk management committees**		
Yes	86	28.4
No	217	71.6
**Training in patient safety**		
Yes	106	35
No	197	65

**Table 2:** Comparison of the 8 dimensions of the two ORMAQ versions.

	Group 1 (n = 10) Original Version (English)				Group 2 (n = 10) Translated Version (French)				F	*p*-Value	R (Pearson)
Dimensions	Mean	SD	Min	Max	Mean	SD	Min	Max			
Leadership structure	12.2	2.57	8	15	12.3	2.54	8	16	0.008	0.931	0.958 **
Confidence-assertion	19.4	2.63	15	24	19.3	2.54	15	23	0.007	0.932	0.993 **
Information sharing	12.8	2.39	9	16	12.8	2.04	10	16	0	1	0.899 **
Stress and fatigue	32.7	5.63	22	42	32.7	5.35	23	42	0	1	0.983 **
Teamwork	27	3.29	21	32	26.7	3.26	21	32	0.42	0.84	0.989 **
Work values	32.6	3.59	26	38	32.3	3.77	26	38	0.33	0.858	0.993 **
Error/procedural compliance	13.2	3.19	8	18	12.9	3.14	8	18	0.45	0.835	0.988 **
Organizational climate	13.3	4.29	6	20	13.1	4.33	6	20	0.11	0.919	0.995 *

* *p* < 0.05; ** *p* < 0.001; SD—standard deviation; Min—Minimum; Max—Maximum.

**Table 3:** Principal Component Analysis.

Rotated Component Matrix ^a^								
	Factors							
	F1	F2	F3	F4	F5	F6	F7	F8
**Confidence-Assertion**								
Q1	**0.790**							
Q14	**0.712**							
Q32	**0.803**							
Q34	**0.713**							
Q36	**0.572**							
Q38	**0.472**							
Q60	**0.581**							
**Leadership structure**								
Q3		**0.803**						
Q10		**−0.683**						
Q27		**0.801**						
Q42		**0.743**						
Q50		**0.765**						
**Information Sharing**								
Q12			**0.653**					
Q13			**0.756**					
Q16			**0.732**					
Q19			**0.765**					
**Errors/procedural compliance**								
Q29				**−0.753**				
Q33				**0.692**				
Q37				**0.796**				
Q41				**0.577**				
Q53				**0.752**				
Q59				**0.549**				
**Organizational climate**								
Q2					**0.683**			
Q24					**0.659**			
Q30					**0.759**			
Q47					**0.705**			
Q57					**0.660**			
Q58					**0.642**			
**Work values**								
Q6						**−0.759**		
Q7						**0.705**		
Q9						**0.664**		
Q15						**0.660**		
Q20						**0.632**		
Q23						**0.557**		
Q26						**0.549**		
Q28						**0.510**		
Q35						**0.488**		
Q40						**0.482**		
Q52						**0.477**		
**Stress and fatigue**								
Q4							**0.423**	
Q5							**0.517**	
Q8							**0.829**	
Q11							**0.512**	
Q21							**0.426**	
Q39							**0.619**	
Q43							**0.516**	
Q45							**0.803**	
Q46							**0.404**	
Q49							**0.601**	
Q51							**0.618**	
Q55							**0.705**	
**Teamwork**								
Q17								**0.552**
Q18								**0.509**
Q22								**0.509**
Q25								**0.761**
Q31								**0.763**
Q44								**0.688**
Q48								**0.483**
Q54								**0.613**
Q56								**0.803**

Extraction Method: Principal Component Analysis. Rotation Method: Varimax with Kaiser Normalization. ^a^: Rotation converged in 9 iterations. Loadings < 0.30 suppressed. Bold values indicate primary factor loading. F: Factor.

**Table 4:** Internal consistency and test–retest reliability of the French ORMAQ.

Dimension	Cronbach’s Alpha	McDonald’s Omega	ICC (95% CI)
Leadership structure	0.773	0.81	0.90 (0.75–0.96)
Confidence–assertion	0.829	0.84	0.85 (0.62–0.94)
Information sharing	0.742	0.78	0.77 (0.42–0.90)
Stress and fatigue	0.654	0.82	0.95 (0.88–0.98)
Teamwork	0.891	0.89	0.75 (0.37–0.90)
Work values	0.766	0.79	0.90 (0.74–0.96)
Error/Procedural compliance	0.597	0.75	0.93 (0.82–0.97)
Organizational climate	0.621	0.75	0.93 (0.83–0.97)
Overall scale	0.842	0.98	0.96 (0.92–0.98)

ICC: Intraclass correlation, CI: Confidence Interval.

**Table 5:** Participants’ attitudes to the different ORMAQ dimensions.

Scores of PSC Domains	Mean ± SD	Min–Max	Strongly Disagree/Disagree n (%)	Neutral n (%)	Agree/Strongly Agree n (%)
**D1: Leadership Structure**	**3.82 ± 0.71**	**1.8–5**			
Senior staff should encourage questions from junior medical and nursing staff during operations if appropriate	4.89 ± 0.42	3–5	8 (2.6)	8 (2.6)	287 (94.8)
Doctors who encourage suggestions from Operating Theatre team members are weak leaders	2.00 ± 1.10	1–5	202 (66.7)	50 (16.5)	51 (16.8)
Successful Operating Theatre management is primarily a function of the doctor’s medical and technical proficiency	3.30 ± 1.25	1–5	104 (34.3)	49 (16.2)	150 (49.5)
Leadership of the Operating Theatre team should rest with the medical staff	3.45 ± 1.20	1–5	89 (29.4)	54 (17.8)	160 (52.8)
There are no circumstances where a junior team member should assume control of patient management	3.40 ± 1.22	1–5	97 (32)	48 (15.8)	158 (52.2)
**D2: Confidence-Assertion**	**3.75 ± 0.68**	**2.0–5**			
The senior person, if available, should take over and make all decisions in life threatening emergencies	4.25 ± 0.95	1–5	28 (9.3)	27 (8.9)	248 (81.8)
Junior Operating Theatre team members should not question the decisions made by senior personnel	3.15 ± 1.30	1–5	92 (30.4)	74 (24.4)	137 (45.2)
If I perceive a problem with the management of a patient, I will speak up, regardless of who might be affected.	4.30 ± 0.88	2–5	26 (8.6)	34 (11.2)	243 (80.2)
In critical situations, I rely on my superiors to tell me what to do	3.95 ± 1.05	1–5	33 (10.9)	44 (14.5)	226 (74.6)
I sometimes feel uncomfortable telling Operating Theatre members from other disciplines that they need to take some action	3.05 ± 1.35	1–5	109 (36)	66 (21.8)	128 (42.2)
Team members should not question the decisions or actions of senior staff except when they threaten the safety of the operation	3.75 ± 1.10	1–5	69 (22.8)	48 (15.8)	186 (61.4)
I always ask questions when I feel there is something I don’t understand	4.70 ± 0.60	2–5	10 (3.3)	24 (7.9)	269 (88.8)
**D3: Information Sharing**	**4.12 ± 0.63**	**2.2–5**			
A regular debriefing of procedures and decisions after an Operating Theatre session or shift is an important part of developing and maintaining effective team co-ordination	4.45 ± 0.76	2–5	11 (3.6)	20 (6.6)	272(89.8)
Team members in charge should verbalise plans for procedures or actions and should be sure that the information is understood and acknowledged by others	4.35 ± 0.80	2–5	11 (3.6)	30 (9.9)	262 (86.5)
I am encouraged by my leaders and co-workers to report any incidents I may observe	4.05 ± 1.00	1–5	41 (13.6)	27 (8.9)	235 (77.5)
The pre-session team briefing is important for safety and for effective team management	4.25 ± 0.85	2–5	13 (4.3)	35 (11.6)	255 (84.1)
**D4: Stress and Fatigue**	**3.41 ± 0.74**	**1.7–5**			
Even when tired, I perform effectively during critical phases of operations	3.90 ± 1.05	1–5	59 (19.5)	35 (11.6)	209 (68.9)
We should be aware of, and sensitive to, the personal problems of other team members	4.05 ± 0.95	1–5	29 (9.6)	55 (18.1)	219 (72.3)
I let other team members know when my workload is becoming (or is about to become) excessive	4.20 ± 0.85	2–5	18 (6)	34 (11.2)	251 (82.8)
My decision making is as good in emergencies as it is in routine situations	3.70 ± 1.10	1–5	52 (17.2)	53 (17.5)	198 (65.3)
I am more likely to make errors in tense or hostile situations	3.20 ± 1.25	1–5	87 (28.7)	71 (23.4)	145 (47.9)
I am less effective when stressed or tired	3.75 ± 1.10	1–5	66 (21.8)	43 (14.2)	194 (64)
My performance is not adversely affected by working with an inexperienced or less capable team member	3.25 ± 1.20	1–5	86 (28.4)	68 (22.4)	149 (49.2)
Team members should monitor each other for signs of stress or tiredness	4.15 ± 0.90	2–5	17 (5.7)	54 (17.8)	232 (76.5)
I become irritated when I have to work with inexperienced medical staff	3.30 ± 1.20	1–5	75 (24.8)	67 (22.1)	161 (53.1)
A truly professional team member can leave personal problems behind when working in the Operating Theatre	4.10 ± 0.95	2–5	29 (9.6)	31 (10.2)	243 (80.2)
Team members should feel obligated to mention their own psychological stress or physical problems to other Operating Theatre personnel before or during a shift or assignment	3.60 ± 1.15	1–5	50 (16.5)	81 (26.7)	172 (56.8)
Personal problems can adversely affect my performance	3.50 ± 1.20	1–5	75 (24.7)	66 (21.8)	162 (53.5)
**D5: Teamwork**	**4.05 ± 0.66**	**2.3–5**			
The only people qualified to give me feedback are members of my own profession	3.40 ± 1.25	1–5	85 (28)	44 (14.5)	174 (57.5)
It is better to agree with other Operating theatre team members than to voice a different opinion	3.45 ± 1.20	1–5	86 (28.4)	42 (13.9)	175 (57.7)
The doctor’s responsibilities include co-ordination between his or her work team and other support teams	4.05 ± 0.95	2–5	26 (8.6)	50 (16.5)	227 (74.9)
Operating Theatre team members share responsibilities for prioritising activities in high workload situations	4.35 ± 0.80	2–5	12 (3.9)	26 (8.6)	265 (87.5)
I enjoy working as part of a team	4.50 ± 0.75	2–5	13 (4.3)	18 (5.9)	272 (89.8)
To resolve conflicts, team members should openly discuss their differences with each other	4.20 ± 0.85	2–5	19 (6.3)	42 (13.9)	242 (79.8)
All members of the Operating Theatre team are qualified to give me feedback	3.00 ± 1.30	1–5	128 (42.3)	61 (20.1)	114 (37.6)
The concept of “all Operating theatre personnel working as a team” does not work at this hospital	3.05 ± 1.25	1–5	107 (35.3)	86 (28.4)	110 (36.3)
Effective Operating Theatre team co-ordination requires members to take into account the personalities of other team members	4.30 ± 0.80	2–5	16 (5.3)	44 (14.5)	243 (80.2)
**D6: Work Values**	**4.18 ± 0.59**	**2.5–5**			
Senior staff deserve extra benefits and privileges	3.45 ± 1.15	1–5	61 (20.1)	84 (27.7)	158 (52.2)
I do my best work when people leave me alone	4.05 ± 0.95	1–5	24 (7.9)	39 (12.9)	240 (79.2)
It bothers me when others do not respect my professional capabilities	4.15 ± 0.90	1–5	17 (5.6)	41 (13.5)	245 (80.9)
I try to be a person that others will enjoy working with	4.35 ± 0.80	2–5	18 (6)	22 (7.2)	263 (86.8)
It is important that my competence be acknowledged by others	4.10 ± 0.90	2–5	25 (8.3)	46 (15.2)	232 (76.5)
I value compliments about my work	4.05 ± 0.95	1–5	17 (5.6)	49 (16.2)	237 (78.2)
As long as the work gets done, I don’t care what others think of me	3.85 ± 1.05	1–5	43 (14.2)	48 (15.8)	212 (70)
A good reputation in the Operating Theatre is important to me	4.25 ± 0.85	2–5	17 (5.6)	37 (12.2)	249 (82.2)
I value the goodwill of my fellow workers- I care that others see me as friendly and co-operative	4.10 ± 0.90	2–5	31 (10.2)	39 (12.9)	233 (76.9)
It is an insult to be forced to wait unnecessarily for other members of the Operating Theatre team	3.70 ± 1.10	1–5	46 (15.2)	57 (18.8)	200 (66)
In the Operating Theatre, I get the respect that a person of my profession deserves	3.95 ± 1.00	1–5	28 (9.2)	49 (16.2)	226 (74.6)
**D7: Error/Procedural Compliance**	**3.12 ± 0.77**	**1.5–5**			
Errors are a sign of incompetence	2.70 ± 1.30	1–5	166 (54.8)	61 (20.1)	76 (25.1)
I am ashamed when I make a mistake in front of other team members	3.10 ± 1.25	1–5	128 (42.3)	61 (20.1)	114 (37.6)
Procedures and policies are strictly followed in our Operating Theatre	3.00 ± 1.20	1–5	111 (36.6)	84 (27.7)	108 (35.7)
Mistakes are handled appropriately in this hospital	2.95 ± 1.20	1–5	109 (36)	102 (33.7)	92 (30.3)
Human error is inevitable	4.10 ± 0.95	2–5	50 (16.5)	49 (16.2)	204 (67.3)
Team members frequently disregard rules or guidelines (e.g., handwashing, treatment protocols/clinical pathways, sterile field) developed for our Operating Theatre	3.10 ± 1.25	1–5	121 (39.9)	65 (21.5)	117 (38.6)
**D8: Organizational Climate**	**3.28 ± 0.81**	**1.6–5**			
The department provides adequate, timely information about events in the hospital which might affect my work	3.20 ± 1.20	1–5	89 (29.4)	86 (28.4)	128 (42.2)
Working in this hospital is like being part of a large family	3.75 ± 1.05	1–5	41 (13.5)	67 (22.1)	195 (64.4)
Departmental leadership listens to staff and cares about our concerns	2.80 ± 1.20	1–5	154 (50.8)	82 (27.1)	67 (22.1)
I am proud to work for this hospital	4.05 ± 0.95	2–5	37 (12.2)	65 (21.5)	201 (66.3)
I like my job	4.70 ± 0.65	2–5	10 (3.3)	13 (4.3)	280 (92.4)
I am provided with adequate training to successfully accomplish my job	3.45 ± 1.10	1–5	86 (28.4)	58 (19.1)	159 (52.5)

n—Frequency; %—Percentage; SD—standard deviation; Min—Minimum; Max—Maximum; Bold values indicate dimensions’ scores.

## Data Availability

The data that support the findings of this study are available from the corresponding author upon reasonable request due to ethical and confidentiality restrictions.
